# Renal hilar block predicts long-term success of renal auto-transplantation for loin pain hematuria syndrome

**DOI:** 10.1007/s11255-019-02143-z

**Published:** 2019-04-11

**Authors:** Jeffrey Campsen, Mitchell R. Bassett, Ryan O’Hara, Robin D. Kim, Eryberto Martinez, Rulon Hardman, Jeremy B. Myers, Blake Hamilton

**Affiliations:** 1 Division of Transplantation and Advanced Hepatobiliary Surgery, Department of Surgery, University of Utah School of Medicine, 30 N 1900 E, Salt Lake City, UT USA; 2 Division of Urology, Department of Surgery, University of Utah School of Medicine, Salt Lake City, UT USA; 3 Section of Interventional Radiology, Department of Radiology and Imaging Sciences, University of Utah School of Medicine, Salt Lake City, UT USA

**Keywords:** Renal, Loin pain, Auto-transplant

## Abstract

**Purpose:**

In patients with loin pain hematuria syndrome (LPHS), a response to percutaneous renal hilar blockade (RHB) and a multidisciplinary team (MDT) evaluation predicts patient’s potential renal auto-transplantation (RAT) success.

**Methods:**

A pain assessment was performed using a 0–10 numeric pain rating scale prior to a percutaneous RHB under CT guidance. If the pain score was reduced > 50% immediately after the RHB, patients were evaluated for RAT by a MDT. Pre-operative and 1-year post-operative quality-of-life surveys were administered to each RAT patient.

**Results:**

43 LPHS patients were referred for RHB. Of the 38 patients who received a RHB, 31 had > 50% reduction in pain scores. Pre- and post-RHB mean pain scores were 6/10 and 0.7/10, respectively, in patients who had > 50% reduction in pain. 22 of the patients who responded favorably then proceeded to RAT. Twelve patients had at least 1-year follow-up after RAT. All patients had a meaningful decrease in their pain. Mean pain score at 1 year was 0.8/10 for an 85% overall reduction in pain. 92% of patients experienced a ≥ 50% reduction in pain at 1 year. Mean Beck Depression Inventory (BDI) score (0–66) 1 year after RAT decreased from 25.2 pre-op (moderate depression) to 12.8 post-op (minimal depression).

**Conclusions:**

A MDT approach utilizing a RHB should be considered as a tool to select appropriate LPHS patients for RAT to achieve long-term success in reducing chronic pain and depression while increasing quality of life.

## Introduction

Loin pain hematuria syndrome (LPHS) was first described in 1967 as severe unilateral or bilateral flank pain with gross or microscopic hematuria [1]. It is a diagnosis of exclusion but many patients have a past urologic history of upper tract obstruction [2]. While many theories exist as to causation, an initial insult such as a kidney stone can create a cascade of chronic pain which can persist long after the obstruction is resolved [2–5]. Once diagnosed, therapies for treatment focus on ablation of the renal nervous system [6–8].

Therapies to treat the chronic pain are percutaneous regional nerve block, surgical sympathectomy, renal capsulotomy, vascular pedicle denervation, ureterolysis, nephrectomy, and renal autotransplantation (RAT) [2]. The evolution of these therapies has shown that denervation of the affected kidney can be effective; however, without removal of the organ, the pain cycle is not adequately and completely disrupted [2]. Previous reports describe RAT as an encouraging therapy for LPHS with reported successful pain relief ranging between 25 and 88% [9–13].

One reason for failure of this therapy is the difficultly of establishing the correct diagnosis [5]. We propose that in patients with classic symptoms of LPHS diagnosed by a senior urologist, a response to percutaneous renal hilar blockade (RHB) can help to predict when a patient should be referred for RAT. If a patient responds to this diagnostic test, then the MDT can proceed with RAT by a transplant surgeon that is part of an established and busy live donor kidney transplant program.

## Methods

We defined LPHS as chronic, unbearable flank pain with or without hematuria in the absence of an infection, nephrolithiasis, or upper tract obstruction. A multidisciplinary team (MDT) was created consisting of an urologist, interventional radiologist, and transplant surgeon to evaluate and treat LPHS. The protocol started with all referrals filtered through our senior urologist to rule out pathology or other causes of pain. After a diagnosis of LPHS was established, all patients were then referred to interventional radiology for a RHB.

**Fig. 1:**
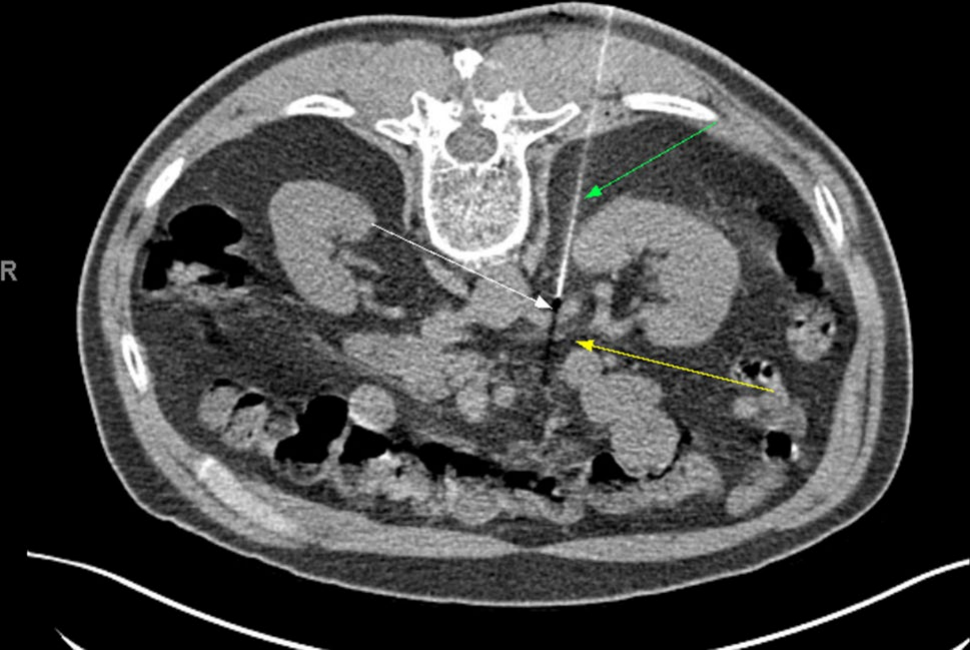
Renal hilar block under CT guidance. Green arrow—needle, white arrow—renal artery, yellow arrow—renal vein

The interventional radiologist evaluated patient pain at the time of the procedure. Blocks were performed using the same protocol. Pain was graded by the patient on a Visual Analog Scale (VAS) from 0 to 10 where 10 is worst pain and 0 is no pain. Patients were positioned prone on a CT table. The renal artery was identified by non-contrast CT (Fig. 1). After administration of subcutaneous lidocaine, a 21-G needle was advanced adjacent to the ipsilateral renal artery. Aspiration was performed to confirm that the needle was not intra-arterial. A 50/50 mixture of 2% lidocaine and 0.5% bupivacaine was administered with total volume of 20 mL. Five patients had additional administration of 40-mg triamcinolone as would be performed in an epidural steroid injection, to see if prolonged pain relief could be provided. Post procedure pain was then queried 10 min after the block was performed, allowing the patient to stand and sit, as what usually exaggerated the patient’s symptoms. A post-procedure pain assessment was then performed. If the pain score was reduced > 50% immediately after the RHB, then s/he was referred to the transplant surgeon for surgical planning. Patients were observed for 30 min after the procedure and then discharged home the same date.

CT angiography was performed using the University of Utah’s live kidney donor protocol prior to the visit for surgical planning. A quality-of-life assessment was performed by a research assistant at the time of surgical consultation and 1 year after RAT. This questionnaire was developed by co-authors through literature reviews to capture necessary outcome measures using self-reported measures of pain and quality of life (QoL), and the Medical Outcomes Study Questionnaire Form 36 Health Survey (SF-36) before and after RAT [14]. The internal development of the questionnaire was a necessity due to the lack of a standardized set of questions geared specifically to this patient population. In addition, patients were asked to fill out the Beck Depression Inventory (BDI) Scale, an internationally recognized instrument for assessing the presence and severity of depression symptoms [15].

## Results

Between 2013 and 2017, 43 new patient encounters were recorded for LPHS in our institution’s urology clinic, and a RHB was recommended in all cases. 38 patients ultimately underwent RHB (two patients proceeded to RAT without RHB, one RHB was denied by insurance, one chose a simple nephrectomy, and one chose treatment through a chronic pain clinic). Mean patient age was 35.8 years. 66% of the patients were female. 63% of the blocks were performed on the right. No major complications were encountered from the hilar block. However, bleeding is the main concern for possible complication. Infection does not appear to be a risk, and antibiotics are not given for the procedure. One patient had a horseshoe kidney, and two renal arteries were targeted; a block was performed at each location. In one patient, the genitofemoral nerve was also affected by the block causing transitory inguinal pain.

Of the 38 patients who received a RHB, 82% of them had > 50% reduction in pain. Pre-RHB mean pain score was 6.0 ± 2.0 with post-RHB pain score being 1.1 ± 1.5. Mean duration of chronic pain prior to RAT was 3.6 ± 7.6 years. Mean pain reduction was 78% for all RHBs. Among those patients who had a < 50% reduction in pain from the RHB, there was a reduction in pain from a mean of 5.1 down to 3.0 accounting for a mean pain decrease of only 34%. Twenty-two (71%) of the patients who responded to the RHB then proceeded to RAT. Nine (29%) patients did not proceed to RAT: one was denied by insurance, four chose alternative therapies (simple nephrectomy-3, intrathecal pump-1), two were not appropriate surgical candidates (horseshoe kidney, solitary kidney in morbidly obese patient with three renal arteries), and two were lost to follow-up. All patients who underwent a RAT had a successful surgery defined by graft function which remains at 100% for all patients. All surgeries were started laparoscopically but two had to be converted to open. Mean operative time was 4 h 58 min. 29% patients had been diagnosed with depression. 74% were taking chronic pain medications. Pain was described as burning by 8 (35%), aching by 17 (74%), stabbing by 21 (91%), and dull by 15 (65%).

**Fig. 2:**
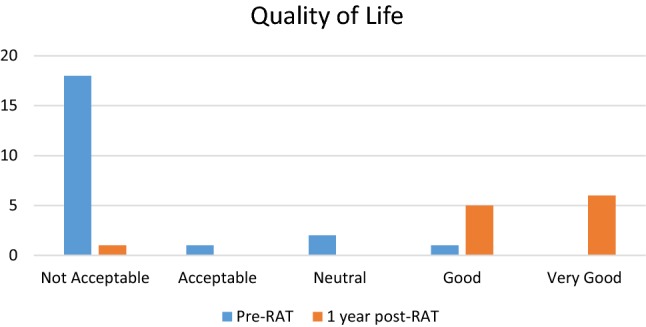
Patient reported quality of life prior to RAT (*n* = 22) and 1-year post-RAT (*n* = 12)

**Fig. 3:**
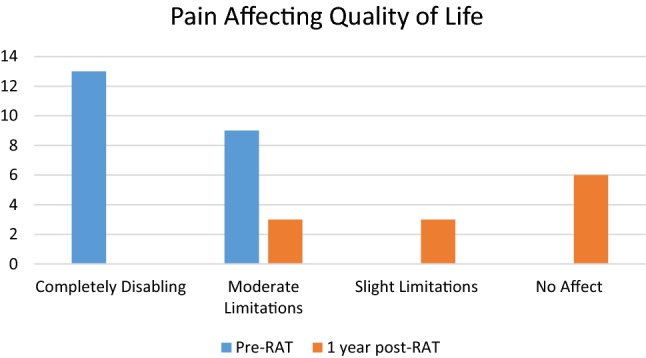
Patient reported effect of pain affecting quality of life prior to RAT (*n* = 22) and 1-year post-RAT (*n* = 12)

**Fig. 4:**
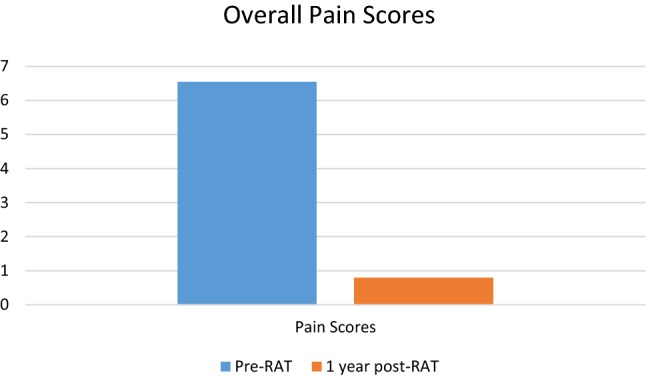
Patient reported pain scores prior to RAT (*n* = 22) and 1-year post-RAT (*n* = 12)

Of the 22 patients who received RAT, 55% patients have at least 1-year follow-up after surgery. One-year pain assessment was 0.8 ± 1.2 with an overall pain decrease of 85%. All 12 patients had a sustained improvement in their pain, while 92% had a 50% or greater improvement. Mean BDI score (0–66) 1 year after RAT decreased from 25.2 pre-op (moderate depression) to 12.8 post-op (minimal depression). There were significant shifts in quality of life and pain affecting quality of life (see Figs. 2, 3, 4). 92% said that RAT was very successful in dealing with symptoms and 92% said that they would undergo the procedure again. 92% said they would recommend the procedure. Pain 1 year after RAT was described as non-existent in 7 (58%), aching in 4 (33%), and dull in 2 (17%).

## Discussion

Successful RAT for LPHS relies on the appropriate diagnosis. We report an effective approach in screening potential candidates for RAT through a RHB using a MDT approach developed at a tertiary referral center. We believe that LPHS is initially caused by the inflammation from kidney stones. When the stones are absent, the pain remains without any other anatomic cause present. This chronic pain is caused by ongoing stimulation of the nerves surrounding the renal hilum. Previous experience has shown that nephrectomy will effectively eliminate this type of pain. The RHB simulates nephrectomy by anesthetizing the nerves temporarily, thus predicting the success of nephrectomy followed by RAT.

In the literature, the success rate of RAT is unacceptably low given the magnitude of the operation [9, 10]. Using our approach, our success rate for patients experiencing a lasting decrease in their chronic flank pain is 100% at 1 year. While 42% of patients still experienced pain on the affected side, the severity was decreased by 85%. Moreover, the quality of life of these patients dramatically improved due to removing the limitations placed on them by their chronic pain. The cohort went from being moderately depressed as measured by the BDI to only minimally depressed 1 year after the transplant. We believe that patients described in the literature who failed to see any improvement in their pain after RAT could have been better vetted by performing a provocative RHB and getting a thorough work-up by all three specialists. Another key to success is a transplant program that has a high-volume live donor kidney transplant program with excellent results as defined by the Scientific Registry of Transplant Recipients (SRTR). UTOP OPO-Specific Report, 2018. https://www.srtr.org/reports-tools/opo-specific-reports/opo?code=UTOP. Accessed 03/26/2018).

One hypothesis our group entertained is if RHB could be used as a bridge to surgery, similar to joint injections prior to arthroplasty. Adding triamcinolone to the injection was tested in five patients. No patients described sustained reduction in their pain beyond the half-life of the bupivacaine; so the steroid addition was discontinued. RHB is a one-time treatment for diagnosis only. Our theory is that any insult to the kidney can create ongoing pain. Furthermore, the block only lasts between 12 and 48 h. A limitation of this study is the small number of patients with a 1-year follow-up. Unfortunately, we do not have enough patients at time of the manuscript creation to power any statistics beyond what is presented. While our 1-year experience is encouraging, we will continue to follow these patients for 5- and 10-year outcomes which are not available at this time.

In conclusion, RAT should be considered in the appropriate population after other disease processes have been ruled out by a MDT. RHB is a tool for the team to define LPHS patient’s appropriateness for RAT. This study confirms that when this protocol is used, RAT can achieve a dramatic reduction in long-term pain allowing for a significant reduction in chronic depression and increase in quality of life.
